# The *BRCA1* c.4096+3A>G Variant Displays Classical Characteristics of Pathogenic *BRCA1* Mutations in Hereditary Breast and Ovarian Cancers, But Still Allows Homozygous Viability

**DOI:** 10.3390/genes10110882

**Published:** 2019-11-01

**Authors:** Adalgeir Arason, Bjarni A Agnarsson, Gudrun Johannesdottir, Oskar Th Johannsson, Bylgja Hilmarsdottir, Inga Reynisdottir, Rosa B Barkardottir

**Affiliations:** 1 Department of Pathology, Landspitali, The National University Hospital of Iceland, 101 Reykjavik, Iceland; bjarniaa@landspitali.is (B.A.A.); gudrunj@landspitali.is (G.J.); bylgjahi@landspitali.is (B.H.); ingar@landspitali.is (I.R.); rosa@landspitali.is (R.B.B.); 2 BMC (Biomedical Center), Faculty of Medicine, University of Iceland, 101 Reykjavik, Iceland; oskarjoh@landspitali.is; 3 Faculty of Medicine, University of Iceland, 101 Reykjavik, Iceland; 4 Department of Oncology, Landspitali, The National University Hospital of Iceland, 101 Reykjavik, Iceland

**Keywords:** *BRCA1*, VUS, LOH, breast cancer, ovarian cancer, tumorigenesis, Knudson’s two-hit model, cancer risk, homozygous lethality

## Abstract

Mutations in *BRCA1* result in predisposal to breast and ovarian cancers, but many variants exist with unknown clinical significance (VUS). One is *BRCA1* c.4096+3A>G, which affects production of the full-length *BRCA1* transcript, while augmenting transcripts lacking most or all of exon 11. Nonetheless, homozygosity of this variant has been reported in a healthy woman. We saw this variant cosegregate with breast and ovarian cancer in several family branches of four Icelandic pedigrees, with instances of phenocopies and a homozygous woman with lung cancer. We found eight heterozygous carriers (0.44%) in 1820 unselected breast cancer cases, and three (0.15%) in 1968 controls (*p* = 0.13). Seeking conclusive evidence, we studied tumors from carriers in the pedigrees for wild-type-loss of heterozygosity (wtLOH) and *BRCA1*-characteristic prevalence of estrogen receptor (ER) negativity. Of 15 breast and six ovarian tumors, wtLOH occurred in nine breast and all six ovarian tumours, and six of the nine breast tumors with wtLOH were ER-negative. These data accord with a pathogenic *BRCA1*-mutation. Our findings add to the current knowledge of *BRCA1*, and the role of its exon 11 in cancer pathogenicity, and will be of use in clinical genetic counselling.

## 1. Introduction

Genetic predisposition to cancer is most often polygenic with no suggestive family history [1], but sometimes it is hereditary, due to a highly penetrant rare mutation, and this is frequently reflected by a prominent family history of the cancer type and an early age of onset [2]. *BRCA1* (MIM# 113705) is one of two large genes (*BRCA1* and *BRCA2*) most frequently mutated in families with hereditary breast and ovarian cancer syndrome (HBOC) [3,4,5]. In female carriers of a pathogenic *BRCA1* mutation, the average cumulative cancer risk by age 70 is 66% for breast cancer (BC) and 41% for ovarian cancer (OC) [6]. The ratio of OC to BC is associated with the location of the mutation in the gene, and is highest within its central "ovarian cancer cluster" region (OCCR), approximately corresponding to exon 11 [7]. Other genetic and lifestyle/hormonal factors also modify these risks for mutation carriers, exemplified genetically by 26 single nucleotide polymorphisms (SNPs) associated with BC risk, and 11 with OC risk [8]. High-risk kindreds with a *BRCA1* mutation may reflect the accumulation of such modifiers, and tested non-carriers in these families may, therefore, or for other reasons, be at elevated risk, or already be affected (known as phenocopies) [9,10]. In many ways, the *BRCA2* gene/mutations can be described similarly, with high tissue-specific risks, and risk-modifying effects of lifestyle/hormonal factors, SNPs and three OCCRs in the gene [6,7,8,9,10]. Importantly for the scope of this article, *BRCA1* and *BRCA2* are tumor-suppressor genes and, in accordance with Knudson’s two-hit model [11], the genotypes in tumors from mutation carriers typically show loss of heterozygosity (LOH), with preferential loss of the non-mutated or "wild-type" copy (wtLOH) of the gene, and its surrounding chromosomal region [12,13]. *BRCA1*-mutated breast tumors also show a tendency to be pathologically distinguished from other breast tumors with regard to expression of estrogen receptors (ER), as the majority of breast tumors in *BRCA1* carriers are ER-negative, while only about 20–30% of other breast tumors (including *BRCA2*-mutated) are ER-negative [14,15]. For *BRCA1* carriers, however, a later age of onset of BC attenuates the *BRCA1*-characteristic prevalence of ER-negativity [15,16,17].

Genetic testing and clinical genetic counselling, in relation to *BRCA1*/*BRCA2* and other disease genes [18,19], relies on a five-tiered classification system (Classes 1–5), based on the degree of likelihood of the pathogenicity of mutations/variants [20]. Class 3, "uncertain", describes the finding of a variant, frequently termed UV (unclassified variant), or VUS (variant of uncertain significance), which complicates test reporting and disease prevention strategies, and, because such variants are individually rare, their assessments greatly depend on the international collaboration of experts and their submissions to web-accessible databases (see, e.g., [21]). A high proportion of variants tentatively remain as uncertain [22,23] and will have to wait for a refined classification, while more evidence is sought. Conclusions may, predictably, be obstructed by factors such as intermediate clinical effects or reduced penetrance of alleles [20]. Unless this is resolved, the clients and their families receive the same clinical recommendations as when no pathogenic mutations are found. Probability calculations have incorporated multiple lines of evidence, such as direct—from statistical genetic studies (case-control, segregation, family history or other)—and evolutionary conservation or other indirect data [24]. When such data are difficult to obtain, some easier-to-collect data have been considered helpful, e.g., presence of the variant in controls and tumor characteristics, such as LOH [25,26,27], grade and expression of ER [26,27] (overview in [28]).

A Danish study reported a spliceogenic variant of *BRCA1*, named c.4096+3A>G (dbSNP rs80358015, also known as 4215+3A>G and IVS11+3A>G in the literature), in the homozygous state, in a healthy 58-year-old woman from a consanguineous family [29]. No homozygous carriers of a pathogenic *BRCA1* mutation had been reported, and homozygous *BRCA1* mutations were known to result in embryonic lethality in mice [30,31]; furthermore, biallelic *BRCA1* mutations are very rare and lead to severe health problems [32,33,34]. This cast doubt on the previous classification of the c.4096+3A>G variant as pathogenic or probably pathogenic by many experts submitting to the ClinVar website, and its significance is now evaluated by most as uncertain [35]. On the other hand, in Europe and the USA, the variant has been reported in cases of breast or ovarian cancer [36,37,38]. It has also been reported in an HBOC family [39], and in families undergoing clinical diagnostic testing, two of which were of Icelandic origin [35], but the variant is not observed in the large, population-based genomic datasets of ExAC (the Exome Aggregation Consortium) and gnomAD (Genome Aggregation Database) (60,706 and 141,456 individuals, respectively) [40,41], nor in the NHLBI ESP (National Heart, Lung, and Blood Institute, Exome Sequencing Project) richly phenotyped collection (selected for traits of importance for heart, lung and blood disorders) of 7034 individuals (6503 samples covered for rs80358015) [42,43]. Also challenging the clinical neutrality of the variant, an experiment using RT-PCR of leukocyte-RNA from a carrier had shown a disturbed splicing profile, viz. a reduction of the full-length *BRCA1* transcript (at most 50% of the full-length signal strength in controls, and therefore compatible with absence from the variant allele) and simultaneous enhancement of the abundance of two naturally occurring transcripts that lack most or all of exon 11: BRCA1-Δ11q and Δ11 (the former retaining 117 nucleotides from the 5′ end of exon 11) [39]. These two, and one called BRCA1-IRIS [44] (with an open reading frame, keeping exon 11 plus 34 extra triplets from intron 11, but skipping all exons thereafter; not assayed in the above-mentioned RT-PCR study), all seem logical candidates for possible clinical consequences of c.4096+3A>G. They are present in control individuals [45]; the Δ11q protein isoform is seen in cell lines lacking the full-length BRCA1 protein, due to truncating mutations in exon 11 [46]; and BRCA1-IRIS overexpression is found in aggressive breast and ovarian cancers [47,48], introducing it as a drug target [49,50,51]. Although the smaller isoforms may, in part, compensate for the lack of a full-length BRCA1 protein (cf. review [52]—with p53 brought into perspective—and as indicated, in relation to therapeutic resistance in cell experiments highlighting Δ11q [46]), they also have unique functions, some in opposition to full-length BRCA1, and a balanced splicing profile seems to be important for the normal physiological function of BRCA1 (reviewed in [53]). Last year, pathogenic *BRCA1* mutations were identified in the homozygous state in four children with multiple congenital anomalies and severe chromosomal fragility; these mutations notably resulted in the absence of a full-length BRCA1 transcript, but in the presence of Δ11q [54]. Hence, explaining the observed benign consequences of c.4096+3A>G homozygosity is not straightforward [29].

**Figure 1:**
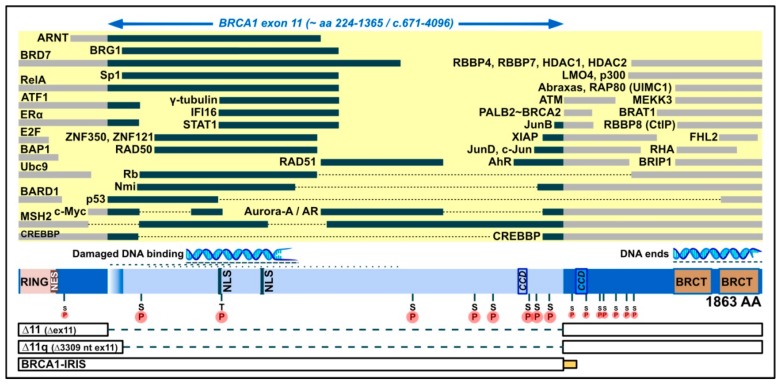
Domain map of the full-length BRCA1 protein (1863 amino acids), emphasizing its middle exon-11-derived region (light blue; bulged if included in Δ11q, but not Δ11 isoforms), which contains two nuclear localization signals (NLS), a coiled-coil domain (CCD), eight serine/threonine phosphorylation spots (pinned S or T on an encircled P), and binding positions for proteins (yellow area, black bars) and for damaged DNA. For comparison, indicated to the left and right of exon 11, are additional protein binding positions (yellow area, grey bars), a position of binding to DNA ends, further serine phosphorylation spots (smaller) and the following domains: RING (which binds, e.g., BARD1), NES (nuclear export signal), BRCT phosphopeptide recognition domains, and a CCD that mediates interaction with PALB2, which bridges to BRCA2. Below, in boxes, are the parts contained in smaller isoforms (connected by broken lines for skipped parts), with the intron-11-derived C-tail of BRCA1-IRIS (bottom) indicated by a small yellow/orange box (not present in full-length BRCA1 or other isoforms).

The BRCA1 protein dates to an early period in the evolution of life [55] and shows a remarkable variety of roles and subcellular localization. As reviewed in [56,57], it is primarily localized in the nucleus, where it functions in maintaining genomic integrity (DNA damage signaling, homologous recombination (HR), homology-directed repair (HDR), and oxidative stress regulation), replication, protein ubiquitination, checkpoint regulation, transcription and chromatin remodeling. It also functions in the cytoplasm, in centrosome regulation, apoptosis and selective autophagy (virophagy and mitophagy). It is found in mitochondria, as well [58]. The joint role of BRCA1 and BRCA2 in HR is thought to explain their common disease phenotype [55], but BRCA1 also has tumor-suppressive effects through other functions (reviewed in [56,57]). It shows sequence-nonspecific (damaged) DNA binding [59,60,61,62] and has many protein partners. Figure 1 (collecting information from reviews [53,57,63,64] and references therein, plus [60,61,62,65,66,67,68,69,70,71]) attempts to give an idea of the switches and plug-ins on the BRCA1 protein that could be specific to a given isoform, highlighting attributes relevant to exon 11. The properties rely inter alia on protein conformation, and are thus not directly predictable from the illustration; as an example, BRCA1-IRIS contains the RING domain but, unlike full-length BRCA1, it seems unable to bind to BARD1 [44]. Likewise, the loss of some properties may be bypassed, as is shown by the ability of Δ11q and Δ11 isoforms to enter the nucleus without any nuclear localization signal (NLS), via binding to BARD1 [72] or Ubc9 [73].

The aim of our study was to test the possible pathogenicity of *BRCA1* c.4096+3A>G. To do this, we genotyped the variant in cases and controls, examined clinical history in the families of identified carriers, and subsequently genotyped key family members. In addition, we analyzed all available tumors from carriers, and present results of LOH and pathological characteristics. Taken together, results indicate that the variant displays classical characteristics of a pathogenic *BRCA1* mutation, both in pedigrees and in tumors. Its population frequency is low (allele frequency in Iceland was ~0.0008). Furthermore, one carrier who developed lung cancer was a homozygote and, since this is the second time homozygosity of this variant in an adult is reported, *BRCA1* c.4096+3A>G must be highly unusual compared to previously analyzed *BRCA1*-mutations.

## 2. Materials and Methods 

DNA was extracted from blood samples and paraffin embedded tissue, as described in previous studies [74]. Quantity of DNA from blood was measured by NanoDrop (Thermo Fisher Scientific), and from paraffin tissue, in the case of LOH analyses, by Qubit Fluorometric Quantification (Thermo Fisher Scientific). For the case–control comparison we used an unselected BC cohort, consisting of 1820 patients (1802 females and 18 males), from whom blood was collected in collaboration with oncologists at Landspitali, the National University Hospital of Iceland, during the period 1987–1999 and at the Research Services Center at Noatun during the period 2002–2009. As controls, we used 1968 samples from a large, population-based control cohort, collected by the Heart Association of Iceland [75]. Taqman assay C_153129896_10 (purchased from Applied Biosystems; current provider Thermo Fisher Scientific) was used to genotype dbSNP rs80358015, using 20 ng of genomic DNA. The reaction was carried out in StepOne Plus Real-Time PCR System (Applied Biosystems). Genotypes of carriers were confirmed by Sanger sequencing (see below). Regarding the possibility of additional genetic variants, or mutations, in chromosomal cis position, relative to the c.4096+3A>G variant, information from the genetic counselling service at the hospital (V. Stefansdottir, personal communication) confirmed this had not been seen when performing next-generation sequencing (NGS) of all exons in *BRCA1* and *BRCA2*. The research was performed in accordance with approval from the Icelandic Data Protection Authority (reference number 2001050523) and the National Bioethics Committee (reference numbers 99/051 and VSN-11-105).

Traced pedigrees were obtained from the Genetic Committee of the University of Iceland, in data files listing individuals and their pedigree relationship. The tracing included great-grandparents’ descendants on both parental sides of each proband, except for one proband, whose pedigrees descended from grandparents. For another proband, an extension of one of her parental sides (tracing back to her grandfather’s grandparents) was available from our earlier studies. The first founders in these pedigrees were born ~1830–1900. A combined list of individuals was sent to the Icelandic Cancer Registry, for collection of clinical data on cancers registered since 1911 (female breast cancers starting in 1911, and all male and female cancers since 1955) [76,77].

Histological immunostaining and HER2-FISH data were collected through routine pathology and gaps filled by staining slides of archived tumors, according to the manufacturer’s protocols (Dako Denmark A/S), using M3643 (dilution 1:20) for ER, M3569 (1:100) for PgR and SK001 (ready to use) for ERBB2. For LOH analyses, DNA was isolated from tumor tissue, which was obtained from paraffin blocks (invasive primary tumors) after selecting areas rich in tumor cells. The proportion of tumor cells in each specimen was estimated by the same investigator and was between 90–100% in 15 samples, ~80% in five and 50–70% in six. Relative allele intensities were compared to those of blood tissue or normal tissue from the same individual. LOH at the *BRCA1* c.4096+3A>G variant site was determined by comparing A and G signals, obtained by Sanger sequencing of the purified products (performed according to standard protocols) of 35 PCR cycles, using 40 ng genomic DNA and primers 5′-GCAGTTCCTTTAACTATACTTGG-3′ (F) and 5′-CAGATGATGAAGAAAGAGGAACG-3′ (R). For analyzing LOH at microsatellite markers, the DNA (40 ng) was amplified for 35 cycles, using PCR primer sequences selected for D17S855 and D17S579 according to the Genome Browser Gateway [78,79], and for THRA1 according to [80]. For both Sanger sequencing and microsatellite fragment analysis, products were run in an ABI 3130xl Genetic Analyzer (Applied Biosystems). They were then analyzed accordingly, with GeneMapper Software 5 (Applied Biosystems) for automatic calling of microsatellite alleles, or Sequencing Analysis Software 6 (Applied Biosystems) and Sequencher 5.4.6 (Gene Codes Corporation) for the alignment of sequences and inspection of electropherograms. For improved resolution of electropherograms, these were printed out and scanned, rather than using screenshots. Signal height comparisons were made using Excel, for A/G signals and for exported GeneMapper files. Figures were drawn and/or processed in Affinity Designer 1.7.0.

## 3. Results

### 3.1. Identification of Probands and Estimation of Allelic Frequencies in Cases and Controls

We genotyped blood-derived DNA samples from 1820 unselected breast cancer (BC) cases and 1968 controls, using the *BRCA1* c.4096+3A>G SNP Taqman assay. All observed carriers had heterozygous genotypes—eight in the BC cohort and three in controls, corresponding to allelic frequencies of 0.0022 and 0.0008, respectively. Fisher’s exact test of this difference was not statistically significant (*p* = 0.13, two-tailed). For decisive statistical comparisons, such low allelic frequencies necessitate larger sample sets than were available to us. We selected the eight carriers among BC cases as probands for tracing pedigrees and family history. Age of onset had the following distribution (using plus signs for contralateral disease at later age): 36 + 60 (also thyroid 65), 37, 40, 43 + 48, 52, 57 (also skin 56), 60 and 69 years. For 1802 female cases in the BC cohort, the subset of 1794 non-carriers had an average age of 57.22 years at diagnosis (95% CI 56.6–57.8), as compared to 49.25 (95% CI 40.9–57.6) for the eight probands.

### 3.2. Pedigrees and Clinical Family History of Probands, and Typing of Affected Relatives 

The pedigrees of six of the eight probands had some overlap. Four probands shared a common large pedigree and were distributed in three family branches, in the fourth and fifth generation. Two other probands were seen to belong to distinct branches (as second cousins) of their shared pedigree. For each of the remaining two probands (including one with the most extensively traced pedigree in this study), neither parental side revealed any familial relation to other probands.

**Figure 2:**
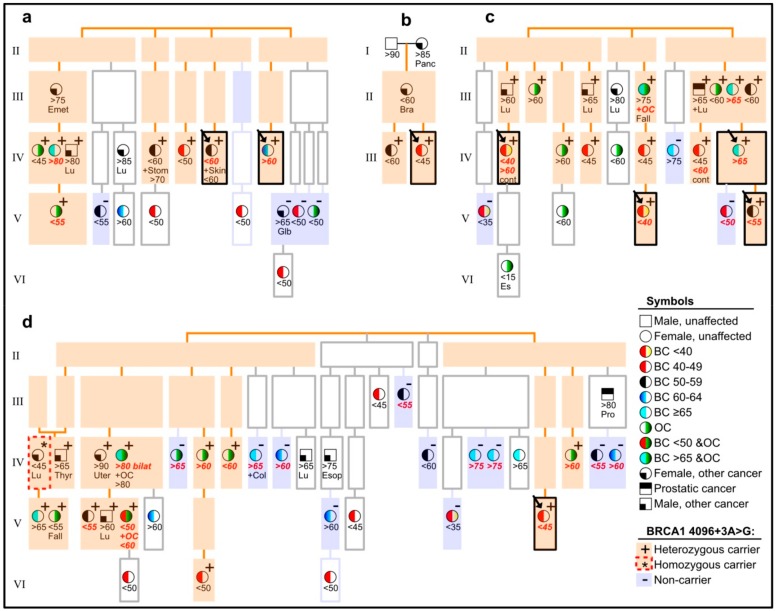
Pedigrees (**a**–**d**) of eight probands (arrows in boxes with black borders), showing cancer-affected individuals and their carrier status, with regard to the *BRCA1* c.4096+3A>G variant, but withholding details on other pedigree members and exact pedigree structures, in order to avoid recognition. Light orange background is used to highlight segregation of the variant, and light violet background highlights cancer-affected non-carriers. Individuals with a white background were not available for genotyping. See explanation of symbols in the lower right corner of the figure (BC = breast cancer, OC = ovarian cancer). Information about approximate age (in years) at diagnosis of cancer is shown below symbols, in accordance with the symbol’s indication of cancer type, plus further details, in case of contralateral breast cancer (cont), fallopian tubes (Fall), endodermal sinus (Es) or cancers other than BC or OC (Bra for brain, Col for colorectal, Emet for endometrial, Esop for esophageal, Glb for gallbladder, Lu for lung, Pro for prostatic, Stom for stomach, Thyr for thyroid, Uter for uterine). Letters in bold red italic denote that the tumor in question was included in analyses of LOH (loss of heterozygosity) and/or histology.

A list of individuals from these pedigrees was matched against Cancer Registry data, and we used the outcome to direct further DNA-sampling and genotyping. Breast and ovarian cancers were observed in a number of family branches. We present the pedigrees in a schematic simplified illustration in Figure 2, in order to help visualize our highlights, with regard to affected carriers vs. non-carriers/phenocopies. This illustration does not include the “opposite-parental” families of probands, where we counted a total of one BC at an age under 60 years, and one OC (any age), when looking for cases amongst 1st, 2nd and 3rd degree relatives of each spouse of a variant-positive parent of a proband. A comparable total count amongst relatives of variant-positive parents (omitting double-counting of overlaps) was nine for BC, under an age of 60 years and eight for OC (cf. Figure 2). In the text below (and in the figure), ages are approximated to the limits of a five-year interval (e.g., <60 meaning age 55–59 years, >70 meaning 70–74 years, etc.).

The pedigrees in Figure 2 vary according to size and information weight. The pedigree in Figure 2a has two branches with probands and two without. The latter can be viewed as having added weight in this study, due to their more distant blood relation to probands. Among seven genotyped cancer-affected members of these two branches (Figure 2a, left half), six were carriers of *BRCA1* c.4096+3A>G, and two of these developed OC. The pedigrees in Figure 2b,c have no such extra branches, while the remaining large pedigree in Figure 2d has three branches, which are even more distantly related to a proband than seen in other families. The most cancer-burdened of these three extra branches (Figure 2d, left part) had a majority of variant-positive cases, and five of these had OC. The other two extra branches of this pedigree (Figure 2d, middle) had relatively few (three) genotyped members, none of whom carried the variant. Other non-carriers were observed in all families, except the small one in Figure 2b. Some of the non-carriers were distantly related to identified carriers (cf. Figure 2a, in generation V) while two were first-degree relatives of affected carriers (Figure 2c, generation V, to the right, and Figure 2d, in generation IV, to the left). The non-carriers were outnumbered by carriers, as seen, e.g., when counting genotyped occurrences of BC diagnosed by age 60 and OC (in any age) in all branches (with or without probands): a total of 38 (14 OC, 24 BC) were found, and 28 (74%; 12 OC, 16 BC) of these occurred in heterozygous carriers of *BRCA1* c.4096+3A>G (Figure 2).

**Figure 3:**
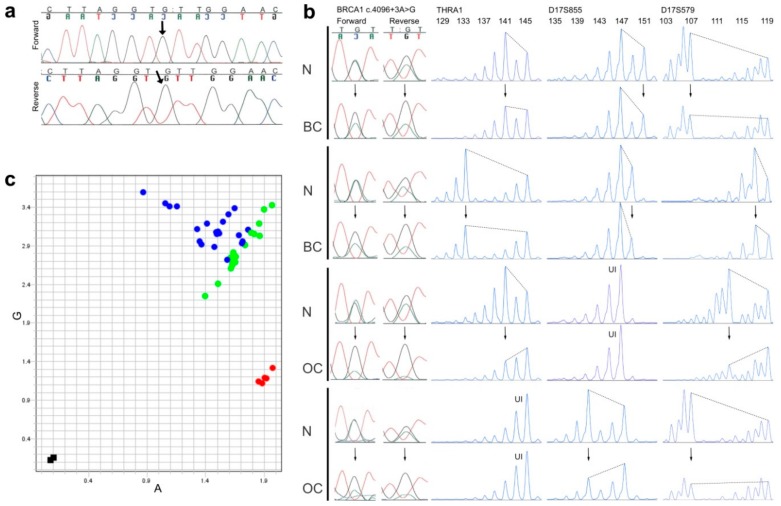
Genotyping and LOH results. (**a**) Sanger sequencing (forward and reverse electropherograms) of the *BRCA1* c.4096+3A>G locus in a homozygous carrier (see Figure 2d, far left in generation IV), the variant signal is indicated by an arrow; (**b**) Examples of pairs of normal (N) and breast (BC) or ovarian cancer tissue (OC) from the same individual, compared for possible loss of heterozygosity in tumor tissue (arrows pointing to altered signal heights in tumors), as analyzed by Sanger sequencing of the *BRCA1* c.4096+3A>G locus (left part, both forward and reverse electropherograms shown) and by PCR-fragment analyses of three microsatellite markers within and flanking the *BRCA1* gene (THRA1, D17S855 and D17S579, with allele sizes in base pairs shown on the top, and UI denoting homozygous genotypes, which were uninformative about LOH); (**c**) An allelic discrimination plot from a *BRCA1* c.4096+3A>G SNP assay of 21 NT-pairs (pairs of matched normal- and tumor-tissue-derived DNA from the same individual) from individuals who were heterozygous for the *BRCA1* c.4096+3A>G variant. Instead of conventional automatic coloring of genotypes provided by the manufacturer’s software, the dots in this graph were manually colored green if they derived from normal tissue and blue if they derived from tumor tissue. Five samples from non-carriers (homozygous for the A-allele) are shown in red, and two negative controls (water) in black.

The most burdened branch in Figure 2d provided an unexpected observation in generation IV (shown farthest left): a woman who was diagnosed with lung cancer at age 40 was genotyped homozygous for the *BRCA1* c.4096+3A>G variant, according to DNA from archived normal thyroid tissue. Autopsy had revealed no other cancers (breasts and ovaries were normal) and clinical records indicated no unusual health problems, but noted that she had many cafe-au-lait spots. Her other parental side (paternal) was unavailable for typing, but no trace was found of the other allele (Figure 3a) and all her four children were heterozygous. The father of the homozygote had geographical origins common to the pedigrees’ top generation founders (see note on geography below). 

### 3.3. A Note on Geographical Distribution in Iceland 

We explored whether the pedigrees could be geographically constricted and saw a clear bias towards a common location in the countryside; the top two generations in the pedigrees lived in an area less than 100 km wide. Inhabitants there constituted ~15% of the Icelandic population around the time the third generation was born. We cannot exclude the possibility that this part of Iceland had (or has) a relatively higher allele frequency, and that this could have biased the genotypes to some extent. But even if all contemporary *BRCA1* c.4096+3A>G carriers lived within that area at the time, one could still expect the local allele frequency to have been low (~0.005, assuming an overall population frequency around 0.0008, like today, but limited to 15% of the population). Aside from this, lower generations (III-VI in Figure 2) were seen to disperse more, with new founders from more widely distributed origins.

### 3.4. Analysis of LOH and Selected Pathological Characteristics 

We performed LOH analyses on all available tumors from carriers (15 BC and six OC samples) and, in addition, we analyzed tumors (four BC and one OC) from non-carriers, if they were closely related (by 1st or 2nd degree) to a genotyped or obligate carrier. A further four BC tumors from non-carriers had histological immunostaining results. For all tumor-derived DNA-samples, a matching blood- or normal-tissue-derived DNA was simultaneously analyzed.

We analyzed the base position of the *BRCA1* c.4096+3A>G variant by Sanger sequencing and looked for allelic imbalance of the A/G signals (Figure 3b, far left). An imbalance exceeding 30% difference of the signals was scored as compatible with LOH. Further to sequencing this gene position, we amplified microsatellite markers, one within *BRCA1* and two flanking the gene, to ascertain whether LOH could have affected another part of the gene, irrespective of the A/G signal ratio (Figure 3b). All instances of imbalance in the A/G spot were compatible with extension to the microsatellite markers, if they had informative genotypes there (not homozygous). No instance of a balanced A/G signal was found with allelic imbalance at the microsatellite markers. All samples with LOH had a reduction in the A-signal compared with normal A/G signals, and a corresponding reduction in microsatellite alleles from the haplotype opposite to the one bearing the *BRCA1* c.4096+3A>G variant (Figure 3b). The preferential loss of the opposite allele can be visualized in Figure 3c, where all the heterozygous tumor DNA, together with their matching normal DNA, were subjected to a *BRCA1* 4096+3A>G SNP assay, and dots in the allelic discrimination plot were subsequently colored differently, according to whether they originated in a tumor or a normal sample (instead of conventional automatic coloring by genotype). All dots deviating from the axis of expected heterozygosity were from tumors and always deviated to the same side (away from control AA genotypes in the graph, and, therefore, closer to representing GG genotypes).

**Figure 4:**
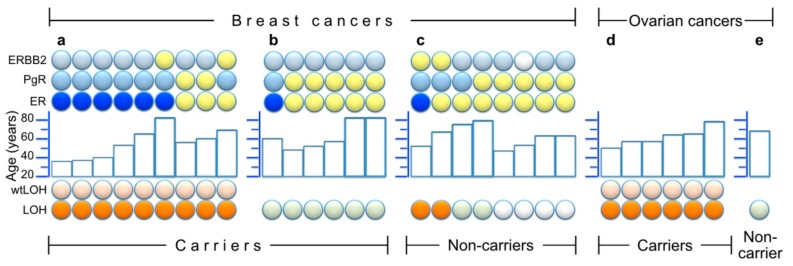
Summary of LOH results and/or histological staining of estrogen-, progesterone- and HER2/Erb-B2 receptors (ER, PgR and ERBB2, respectively) in 30 tumors. Within each partition of the figure, the samples are ordered from left to right by LOH results (bottom symbols: orange for LOH and light orange above that, denoting wtLOH, light green for samples without LOH, and white for unavailable LOH information), and then by ER results (ER line of symbols: blue for ER negative, yellow for ER positive) and finally by age (middle histogram) and other receptor results (top two lines of symbols: light blue = negative, yellow = positive and white = unavailable). (**a**) Nine breast cancers from heterozygous carriers who had wtLOH in their tumors; (**b**) Six breast cancers from heterozygous carriers without LOH; (**c**) Eight breast cancers from non-carriers among close (1st and 2nd degree) relatives of genotyped or obligate carriers (four of which were available for LOH analysis of microsatellite markers); (**d**) Six ovarian cancers from carriers; (**e**) One ovarian cancer from a non-carrier.

Figure 4 summarizes the results of the LOH-analyses for both carriers and non-carriers, and combines these with some clinical (tumor type, age of onset) and pathological (ER/PgR/HER2-immunostaining) information. As shown, nine of 15 BCs in carriers (Figure 4a,b) showed wtLOH, and these nine had a majority of ER-negative breast cancers (six out of nine). This group of ER-negative wtLOH-tumours contains the three lowest-age diagnoses of BC in this study (35–40 years). In the group of six genotype-balanced tumors from carriers (Figure 4b), only one was ER-negative, whereas the other five were ER-positive. As regards BC from non-carriers (Figure 4c), their ER and age distribution resembles that of the genotype-balanced group of carriers. Four of these had been analyzed for allelic imbalance at microsatellite markers and two of them had shown LOH. The LOH results from OC samples showed wtLOH in all six OCs from carriers (Figure 4d), in contrast with perfectly balanced microsatellite genotypes in the non-carrier (Figure 4e).

### 3.5. Instances Where Two Studied Tumors Originated in the Same Person

Three women had more than one tumor analyzed in our study. (1) A proband (Figure 2c, far left branch) first had an early-onset ER-negative BC (age < 40) and later developed a contralateral ER-positive BC at age >60. Both her tumors showed wtLOH. (2) A woman (Figure 2d, generation IV) had bilateral BC at age >80, and both were without LOH and were ER-positive. (3) A woman (Figure 2d, generation V) first had BC at age <50; this was ER-positive and without LOH. She developed OC at age <60 and this tumor showed wtLOH.

## 4. Discussion

In our study, we found many factors in favor of pathogenic classification of *BRCA1* c.4096+3A>G. Firstly, our sample collections showed a higher allele frequency in BC cases (0.22%) than in controls (0.08%). Secondly, we found in our extensively traced pedigrees a number of branches with BC- and OC-affected variant carriers who were distantly related to probands. We even found, in one pedigree, a BC-affected carrier who was an 8th degree relative of the proband (Figure 2d, generation VI). We have extended some pedigrees similarly for other *BRCA1*/*BRCA2* mutations (pathogenic), with fewer instances of distant branches with affected carriers, and more branches with affected non-carriers (unpublished results). Thirdly, a clear majority of breast and ovarian cancers in these pedigrees occurred in carriers as compared to non-carriers. This is most striking for OC, because only two of 14 genotyped OC occurred in proposed phenocopies, but is also notable when considering BC diagnosed by age 60, as there were 16 cases in carriers vs. eight in non-carriers. We do not consider a total of ten proposed phenocopies in the four pedigrees to be suspiciously high in view of the size of these pedigrees and when comparing them to our similarly extended pedigrees, with other *BRCA1*/*BRCA2* (pathogenic) mutations (unpublished results). Fourthly, examination of both parental family sides of each proband revealed only two occurrences of BC (by age 60) or OC (any age) among 1st to 3rd degree relatives of the variant-negative parents, as compared to 17 (nine BC < 60 and eight OC) for the variant-positive parents. Lastly, a majority of breast and ovarian cancer specimens from carriers showed classical signs of *BRCA1*-mutated tumors. These indications have some limitations, such as lack of statistical power (larger sample collections are needed for the comparison of cases and controls) and less certain reference frequencies of the variant in the upper generations of pedigrees, but they all point in the same direction, and agree with a pathogenic *BRCA1* mutation. As stated earlier, *BRCA1* c.4096+3A>G was the only variant identified by exonic NGS of the *BRCA1* and *BRCA2* genes in a carrier (V. Stefansdottir, personal communication), so this is hardly explained by a hidden *BRCA1* mutation cosegregating with the variant.

As the variant here appears to be pathogenic, it is important to evaluate the degree of its cancer risks. As regards our pedigrees, our sampling for genotyping was focused on cancer diagnoses and did not include unaffected relatives (due to the inaccessibility of such samples with informed consent), which would have been preferable in order to calculate a precise estimation of risk. On the other hand, our comparison of cases and controls indicates the risk of BC to be only moderately increased (two- or three-fold). We did not have the means to estimate the variant frequency in OC cases in general, but our pedigrees suggest a considerable OC risk, and all the OC samples from heterozygous carriers in our LOH analysis behaved as classical *BRCA1*-mutated, with respect to wtLOH. With the above indications of intermediate BC risk and high risk of OC, we consider the variant pathogenic, but apparently different from typical class 5 pathogenic variants. *BRCA1* c.4096+3A>G could hardly be considered only a risk-modifying variant. Such variants are known to confer significant increase in risk, but its magnitude is low in each variant and their variability high, and, therefore, they are not expected to cosegregate with a disease in pedigrees. The magnitude and organ specificity of the risk conferred by *BRCA1* c.4096+3A>G remains to be defined in more detail.

As regards our LOH analyses, expecting LOH in tumors may at first glance seem illogical, when considering a variant that has been reported in the homozygous state in a healthy adult. One could regard the homozygous woman as already having “two hits” in her entire cell population. But LOH usually adds damage, by causing a larger part of the gene or chromosome to disappear. We frequently found this in the studied tumors, always affecting the wild-type copy in heterozygotes. This clearly indicates that one copy of the variant, in a state of haploinsufficiency, is not enough for the healthy growth of cells, and is linked to tumor induction or progression. Were it only a question of haploinsufficiency, irrespective of the variant, then the LOH would be expected to affect the normal or variant alleles at random. Since it does not, the variant must have a reduced functionality which, in haploinsufficiency, is ideal for tumor development. However, as long as a nonmalignant cell has two copies of the variant, functionality can be sufficient for normal growth.

Our samples from heterozygous breast tumors included nine with wtLOH and six without any LOH. The latter apparently arose for different reasons, or followed a different path of progression, and tended to occur at a later age. Within the BC group of nine tumors with wtLOH, a clear majority were also ER-negative, and this too reflected the distribution of age of onset; the three youngest cases (35–40 years) were ER-negative, whereas the three ER-positive tumors occurred between 55–70 years. This is in line with seeing ER-negativity less frequently with a higher age of onset in *BRCA1* mutation carriers (cf. [15]). As mentioned in the Results, two of the breast cancers in the group with wtLOH were from the same individual, who first had an early-onset ER-negative BC (age <40), and later developed a contralateral ER-positive BC, at age >60. This coincides with a different hormonal background (pre-/postmenopausal), and, at the same time, it is noteworthy that these two cancers occurred in the same genetic background, and did not differ with respect to wtLOH.

We conclude that the data we have obtained strongly favor the pathogenicity of the *BRCA1* c.4096+3A>G variant. It would be very hard to explain our results if the variant is considered clinically benign. Therefore, with respect to the, now twice-reported, strikingly uncommon occurrences of good health lasting into adulthood, in spite of homozygosity, we propose that further studies and experiments on this mutation, or others affecting the protein in a similar way, may cast light on a specific, tumor-suppressive nature of *BRCA1* that has not yet been recognizable from other *BRCA1* functions. It is important to separate the many *BRCA1* functions and match them to different mutations or modifications of the BRCA1 protein, be it the full-length product or alternatively spliced transcripts. Furthermore, this might help to understand why the OC risk is higher when a mutation in *BRCA1* affects exon 11. We have demonstrated strong effect on OC risk, and note that the variant mainly disturbs the splicing profile of BRCA1. In addition to these points, the occurrence of viable homozygosity, and the moderate risk indications seen in the case/control comparison, may reflect a modifying factor, yet to be identified. The exact degree of splicing will also be of concern in future studies of the variant, but we note that, in the study of Wappenschmidt et al. [39], the full-length product in controls had at least double the intensity of the one in the heterozygous sample. Therefore, the variant allele does not seem to be capable of much expression, if any, of the full-length product. To address this in greater detail, RNA studies of homozygous material would be ideal, but these are unavailable at present.

We hope this study, and our brief review of the BRCA1 protein in Figure 1, will serve to promote new ideas, and further molecular studies of *BRCA1*.

## 5. Conclusions

The *BRCA1* c.4096+3A>G variant is pathogenic and confers an increased risk of both breast and ovarian cancers. Its exact refined classification in the five-tiered classification system may be complicated by the fact that these risks have yet to be precisely estimated, and they need to be studied further with respect to modifying genetic and lifestyle factors. The c.4096+3A>G variant is unique compared to previously identified pathogenic *BRCA1* mutations, because it can be found in the homozygous state in healthy individuals.
